# Comment on “Comparison of BISAP, Ranson, MCTSI, and APACHE II in Predicting Severity and Prognoses of Hyperlipidemic Acute Pancreatitis in Chinese Patients”

**DOI:** 10.1155/2017/1426486

**Published:** 2017-07-12

**Authors:** Yuchen Wang, Bashar M. Attar

**Affiliations:** ^1^Internal Medicine, Cook County Health and Hospitals System, Chicago, IL, USA; ^2^Department of Gastroenterology and Hepatology, Cook County Health and Hospitals System, Chicago, IL, USA

The recently published paper by Yang and colleagues on the comparison of frequently used severity-assessment scores in patients with hypertriglyceridemia-induced acute pancreatitis (HP) provided important information and represented the largest cohort with such focus in this population [1]. We would like to point out several issues that potentially complicated the interpretation of the results presented by the authors.

Our primary concern is that one of the outcomes (severity of HP) was defined by the scores calculated (Ranson, APACHE II, BISAP, and MCTSI scores). The authors then tested each individual score's performance in predicting the outcome. Such an assessment introduced incorporation bias, which occurs when components of an index test also are incorporated in the outcome. Incorporation bias is expected to overestimate test accuracy [2]. A similar issue existed when utilizing the MCTSI score to predict local complications, as both are evaluated by contrast-enhanced computed tomography (CECT). In fact, several findings accounted in MCTSI score were used to define local complications, such as peripancreatic fluid collection and pleural effusion [3]. Of note, the study primarily aimed to compare the results and prediction values of the four scoring systems, for which the methodology applied was adequate. However, for a more precise evaluation of the clinical course of HP, more objective information such as length of stay, readmission rate, and mortality would expectedly serve better as primary endpoints.

One potential limitation of directly applying the existing scoring systems for acute pancreatitis to HP is the lack of consideration for concurrent diabetic ketoacidosis, which is highly prevalent in this population. As Quintanilla-Flores et al. demonstrated in their cohort of 55 HP cases, 14.5% patients (8 cases) had concurrent DKA [4]. On the other hand, in the setting of DKA, hypertriglyceridemia represents 36.4% [5] of acute pancreatitis episodes, significantly higher than the 1 to 4% prevalence in the non-DKA population [5, 6]. The triad of hypertriglyceridemia, acute pancreatitis, and DKA was referred as an “enigmatic triad” by Nair and Pitchumoni to describe the complicated pathophysiology [7]. In the setting of DKA, severity scores can be increased by the following factors: the Ranson score is increased by elevated glucose, base deficit, and fluid sequestration; and, the APACHE II score is increased by low arterial pH. In prior studies, Ranson and APACHE II scores were significantly higher among patients with concurrent DKA, while no difference in clinical course (hospitalization length, delay in PO intake, duration of intravenous insulin infusion) was identified between the two groups [4]. This discrepancy suggested the necessity of designing a scoring system that incorporates the potential presence and influence of DKA, as well as the dynamic clinical response of individual patients.

According to the Revised Atlanta Classification of Acute Pancreatitis [8], moderate–severe acute pancreatitis (MSAP) and severe acute pancreatitis (SAP) are defined based on the presence of transient or persistent organ failure [8]. Therefore, the two existing systems that evaluate organ function might be better poised to determine the severity of HP are the Marshall score and the sequential organ failure assessment (SOFA) score [9, 10].

In conclusion, we congratulate Yang and colleagues on their study, which provided valuable information in regard to frequently used scoring systems to determine the severity of HP. However, concerns of incorporation bias and the lack of consideration of diabetic ketoacidosis in existing scoring systems complicated the interpretation of their findings. Therefore, we propose that it is necessary to design a HP-specific scoring system to better characterize and predict the clinical courses and outcomes of HP.

## References

[1] Yang L., Liu J., Xing Y. (2016). Comparison of BISAP, Ranson, MCTSI, and APACHE II in predicting severity and prognoses of hyperlipidemic acute pancreatitis in Chinese patients. *Gastroenterology Research and Practice*.

[2] Schmidt R. L., Factor R. E. (2013). Understanding sources of bias in diagnostic accuracy studies. *Archives of Pathology & Laboratory Medicine*.

[3] Mortele K. J., Wiesner W., Intriere L. (2004). A modified CT severity index for evaluating acute pancreatitis: improved correlation with patient outcome. *Pancreas*.

[4] Quintanilla-Flores D. L., Rendón-Ramírez E. J., Colunga-Pedraza P. R. (2015). Clinical course of diabetic ketoacidosis in hypertriglyceridemic pancreatitis. *Pancreas*.

[5] Nair S., Yadav D., Pitchumoni C. S. (2000). Association of diabetic ketoacidosis and acute pancreatitis: observations in 100 consecutive episodes of DKA. *The American Journal of Gastroenterology*.

[6] Fortson M. R., Freedman S. N., Webster P. D. (1995). Clinical assessment of hyperlipidemic pancreatitis. *American Journal of Gastroenterology*.

[7] Nair S., Pitchumoni C. S. (1997). Diabetic ketoacidosis, hyperlipidemia, and acute pancreatitis: the enigmatic triangle. *The American Journal of Gastroenterology*.

[8] Talukdar R., Vege S. S. (2015). Revised Atlanta classification of acute pancreatitis. *Prediction and Management of Severe Acute Pancreatitis*.

[9] Marshall J. C., Cook D. J., Christou N. V., Bernard G. R., Sprung C. L., Sibbald W. J. (1995). Multiple organ dysfunction score: a reliable descriptor of a complex clinical outcome. *Critical Care Medicine*.

[10] Vincent J. L., De Mendonça A., Cantraine F. (1998). Use of the SOFA score to assess the incidence of organ dysfunction/failure in intensive care units: results of a multicenter, prospective study. *Critical Care Medicine*.

